# “It unsticks your mind”: Using a musicians’ masterclass to introduce oncology faculty and trainees to the practice of direct observation and coaching

**DOI:** 10.36834/cmej.69892

**Published:** 2020-09-23

**Authors:** Michael Sanatani, Kylea Potvin

**Affiliations:** 1 Department of Oncology, Western University, Ontario, Canada

## Implication Statement

Bringing faculty to a realization of the importance of direct observation is a major task during the transition to competency-based medical education. Musicians generally already endorse a strong coaching culture. We included a live cello masterclass in an oncology faculty and trainee workshop in order to demonstrate coaching and feedback. Based on participant post-event interviews, the musical masterclass was a highly effective catalyst for self-reflection in regards to teaching practices and led to new and revised perspectives on observation and coaching in medicine. With just a musician-trainee, music coach, and faculty moderator, this effective demonstration can be easily replicated.

## Déclaration des Répercussions

Amener le corps professoral à réaliser l’importance de l’observation directe est une tâche majeure à accomplir au cours de la transition vers la formation médicale axée sur les compétences. Habituellement, les musiciens font preuve d’une solide culture de coaching. Nous avons intégré un cours de maître de violoncelle donné en direct à un atelier destiné au corps professoral et résidents d’oncologie afin de démontrer le « coaching » et la rétroaction. En se basant sur les entrevues effectuées après l’événement, le cours de maître de musique a été un catalyseur efficace pour l’auto-réflexion sur les pratiques d'enseignement et a donné lieu à des perspectives nouvelles et ajustées sur l’observation et le « coaching » en médecine. Cette démonstration convaincante, qui ne nécessite qu’un musicien en formation, un « coach » en musique et un modérateur du corps professoral, peut facilement être reproduite.

Direct observation is a prerequisite for assessment in competency-based medical education, and sustained observation of performance has been identified as crucial to the faculty-trainee relationship ^1^ Music and sports have very strong cultures of cyclical observation, feedback, and performance within a faculty-trainee relationship (“coaching”).^2^

Hoping to address our (medical) faculty’s reluctance towards giving constructive feedback and underestimation of observation importance, as well as trainees’ often limited receptivity towards feedback,^3^ we incorporated a classical cello masterclass into a CBME introduction workshop. The study was approved through the Western University Health Sciences Research Ethics Board.

The 40 attendees witnessed a professional cellist work with one of the authors (MS), an amateur cellist. The moderator (KP) then elicited audience observations and facilitated a 45-minute discussion. The audience articulated their observations and how the coaching observed might be transferred to clinical teaching. A video is available at https://www.youtube.com/watch?v=Bi3PEoDdF_g&t=1297s.

Thirteen attendees were interviewed by the authors (Table 1). Thematic analysis^4^ of the post-event interview transcripts by MS and KP indicated that the demonstration

**Figure 1:**
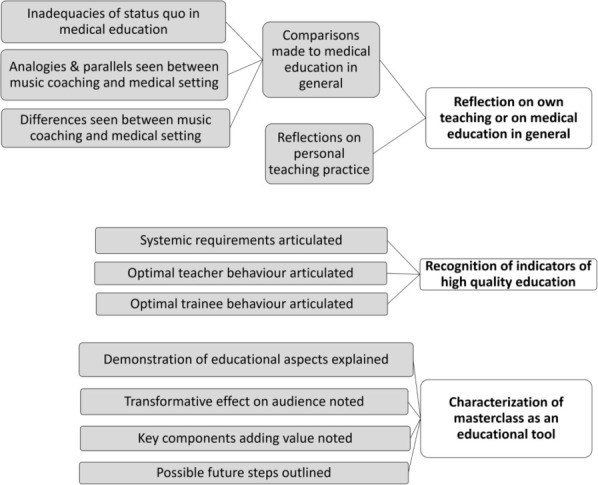
Thematic analysis of post-workshop interviews with faculty and trainees: emergent coding framework

- triggered reflection by the audience on their own teaching/learning habits,
- highlighted indicators of high-quality teaching in general, and
- was an educational tool with a beneficial effect on the audience including changed perspectives on giving and receiving of feedback (Figure 1).

**Table 1:** Semi-structured interview questions post-event

Did you ever participate regularly in an activity involving coaching, such as sports or music? If yes, can you describe your activity and the role you played in it? If yes, do you still do one or more of these activities? Has your role changed over time? When observing the masterclass, did you see any parallels to medical training? If yes, what were the similarities? If not, what made it different? Considering your own past experiences with medical faculty/trainee interactions, did what you observed with the student/musician give you any ideas about how medical education interactions could be made more effective for learning? Could you specify what medical faculty might do differently or emphasize differently than currently? Could you specify what medical trainees might do differently or emphasize differently than currently?

Faculty realized how current teaching practices were limited:

*I think the main issues with our training here is that we aren’t doing enough direct observation and enough coaching. Most of the interactions I have observed would be residents seeing a patient, coming out of the room and just describing what they did or saw with very little feedback*. (Faculty #11)

Both trainees and faculty reported noticing other elements of effective teaching:

***Timelinessof feedback***

*In the moment really struck me. Immediate in time*. …. (Faculty #12)

***Feedback receptivity***

*In…medicine it is all emotional. We all take any criticism as a personal affront …[but] you didn’t take any of it personally you were just making those corrections and it was just a technique change and there was no personal “oh I’m so hurt that she didn’t like the way I played that.”* (Trainee #5)

***Specificity***

*…the advice she gave was very specific and it was very limited. … It was that small little bites with the positive reinforcement but still what needed to be said was said*. (Trainee #5)

Residents in particular felt having a faculty member as the “musician-trainee” in the workshop was helpful:

*I do think this is a nice way to transition the residents …Once you see your attendings getting feedback, then you don’t feel so bad because everyone gets feedback which is critical and important*. (Trainee #1)

We have since repeated this workshop in a Program Directors’ retreat and in a faculty course on coaching. We would encourage other centres to try this, and to consider including medical students^5^ in such workshops (our next step!) in order to introduce a coaching culture as early as possible in their professional development.
